# Effects of parasitic infection and reproduction on corticosterone plasma levels in Galápagos land iguanas, *Conolophus marthae* and *C. subcristatus*

**DOI:** 10.1002/ece3.3077

**Published:** 2017-07-05

**Authors:** Michela Onorati, Giulia Sancesario, Donatella Pastore, Sergio Bernardini, Marilyn Cruz, Jorge E. Carrión, Monica Carosi, Leonardo Vignoli, Davide Lauro, Gabriele Gentile

**Affiliations:** ^1^ Department of Science University Roma Tre Rome Italy; ^2^ Direction of the Galápagos National Park Puerto Ayora, Santa Cruz Island, Galápagos Islands Ecuador; ^3^ Department of Clinical and Behavioural Neurology IRCC S. Lucia Rome Italy; ^4^ Department of Systems Medicine University of Rome Tor Vergata Rome Italy; ^5^ Department of Experimental Medicine and Surgery University of Rome Tor Vergata Rome Italy; ^6^ Galápagos Genetics, Epidemiology and Pathology Laboratory Galápagos National Park & University of Guayaquil Puerto Ayora, Galápagos, Islands Ecuador; ^7^ Department of Biology University of Rome Tor Vergata Rome Italy

**Keywords:** baseline levels, ELISA, glucocorticoids, hemoparasites, *Hepatozoon*, parasitemia, pink iguana, Wolf volcano

## Abstract

In vertebrates, one main feature of stress response is the release of glucocorticoids (corticosterone in reptiles), steroid hormones whose synthesis is regulated by the hypothalamic–pituitary–adrenal axis (HPA). In the Galápagos Islands, populations of land iguanas are differentially impacted by a tick‐transmitted apicomplexan hemoparasite of genus *Hepatozoon*, which could cause diseases and ultimately reduce fitness. Using competitive enzyme‐linked immunosorbent assays (ELISA), we examined baseline plasma corticosterone levels of two syntopic and highly parasitized populations of the land iguana species *Conolophus marthae* and *C. subcristatus* in Wolf volcano (Isabela Island). We also used a poorly parasitized population of *C. subcristatus* from the same island (Bahia Urbina) as a reference. To better interpret the observed glucocorticoids patterns, we simultaneously performed the count of white blood cells (WBCs) in all individuals and investigated the reproductive status of females. We did not find evidence in support of either a positive or negative relationship between the tick load, hemoparasite infection, and glucocorticoid plasma concentration in *C. marthae* and *C. subcristatus* at Wolf volcano. The comparison between parasitized and non‐parasitized sites (V. Wolf and Bahia Urbina) would instead suggest an inverse relationship between corticosterone and parasites. Our findings support association between corticosterone plasma levels and reproduction.

## 1 INTRODUCTION

In recent years, glucocorticoid levels have been increasingly used as physiologic indices of individual and population health (Bonier, Martin, Moore, & Wingfield, 2009; Romero, 2004; Walker, Boersma, & Wingfield,^2005^; Wikelski & Cooke, 2006; Wingfield et al., 1997). Elevated baseline levels have been observed in animals facing both environmental (Foley, Papageorge, & Wasser, 2001) and anthropogenic disturbances (Creel, 1997; Wingfield & Romero, 2001). Generally, high levels of glucocorticoids are related to individuals or populations in worse health status (Bonier et al., 2009).

Glucocorticoids (GCs) are steroid hormones secreted in response to a multiplicity of stressors (Sapolsky, Romero, & Munck, 2000). When an internal and/or external environmental change occurs, the hypothalamic–pituitary–adrenal (HPA) axis stimulates the secretion of GCs by the adrenal glands to help organism in responding to stressful conditions (McEwen & Wingfield, 2003; Wingfield, 2013; Wingfield & Ramenofsky, 1999; Wingfield & Romero, 2001; Wingfield & Sapolsky, 2003; Wingfield et al., 1997). Glucocorticoids are the final product of the HPA axis and participate in the control of homeostasis, activating immediate life‐saving processes (Romero, Dickens, & Cyr, 2009). Normally, short‐term glucocorticoid releases are helpful for individual survival because they stimulate both physiologic and behavioral emergency mechanisms, exclusively oriented to overcome the perturbation (Wingfield & Romero, 2001; Wingfield & Sapolsky, 2003). However, long‐term activation of the stress response with chronically elevated GCs concentrations could be prejudicial. Prolonged elevated concentrations could expose the individual to a long‐term overstimulation of survive mechanisms with consecutive inhibition of many fundamental functions including reproduction, growth, and immunocompetence (Dallman & Bhatnagar, 2001; Dhabhar, 2000; Sapolsky, 1987; Sapolsky et al., 2000; Wingfield et al., 1997). Therefore, persistent high levels are usually detrimental to health, as they may augment the stress‐related disease and pathology (Romero et al., 2009).

In reptiles, corticosterone (CORT) is the primary adrenal glucocorticoid hormone produced to promote advantageous responses against stressful events (Greenberg & Wingfield, 1987; Hanke & Kloas, 1995). Many stressors have been observed to produce effects on CORT levels including physical factors (temperature: Lutterschmidt & Mason, 2009; Telemeco & Addis, 2014; extreme weather events: Romero & Wikelski, 2001) and biotic stressors such as predation (Thaker, Lima, & Hews, 2009), social competition (Comendant, Sinervo, Svensson, & Wingfield, 2003), and especially parasitic infections (Hanley & Stamps, 2002; Sperry, Butler, Romero, & Weatherhead, 2009).

The effects of parasites are energetically demanding (e.g., reduction of growth and long‐term survival; Madsen, Ujvari, & Olsson, 2005), which is why the increase in CORT concentrations is necessary to better cope with the challenges of parasitism (Raouf, Smith, Brown, Wingfield, & Brown, 2006). Although hormonal alterations accompany parasitic infection, the interpretation of stress response using CORT requires always a careful interpretation. In fact, CORT plasma levels vary not only in response to stressor‐dependent factors (duration and intensity), but they are also susceptible to individual‐dependent factors (e.g., reproductive status) which should be considered when GCs are used as physiologic indices of condition in wild populations (Breuner, Wingfield, & Romero, 1999; Moore & Jessop, 2003; Romero, 2002).

The role of CORT during reproduction is very complex. If, on one hand, CORT elevations due to a stress condition shift the individuals into an “emergency life‐history stage” in which they reduce or even abandon their parental activities as reproduction (Greenberg & Wingfield, 1987; Wingfield et al., 1998); on the other hand, an increasing number of studies report positive associations between reproduction and CORT as a result of the involvement of such hormone in the energetically demanding breeding/nesting activities (Rubenstein & Wikelski, 2005; Tyrrell & Cree, 1998), or specifically in oogenic processes (Wilson & Wingfield, 1992). Although a cause–effect link exists between corticosterone and reproduction, the interpretation of such relationship may be difficult, as different factors influence glucocorticoids production.

**Figure 1:**
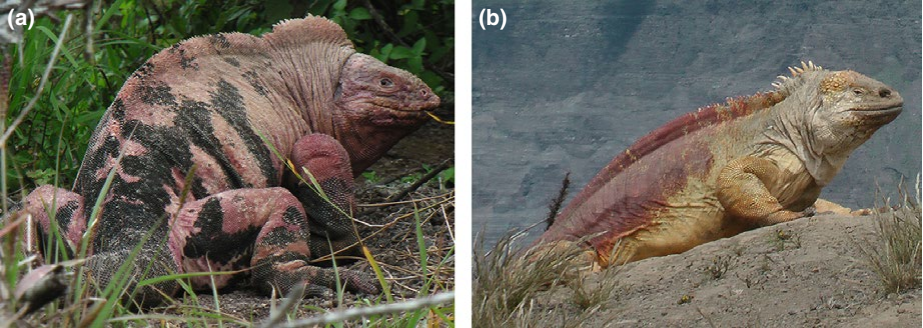
*Conolophus marthae* (A) and *C. subcristatus* (B). Photos G. Gentile

In this study, we related plasma CORT concentration to levels of tick load and hemoparasitic infections in two populations of the two Galápagos land iguana *Conolophus marthae* (*CM*) and *C. subcristatus* (*CS*), syntopic on Wolf volcano, Isabela Island (Gentile & Snell, 2009) (Figure 1). These two populations are highly parasitized by *Hepatozoon* sp. (Apicomplexa: Adeleorina), cosmopolitan blood parasites found in a large number of reptilian hosts (Al‐Ghamdi et al., 2011; Cook, Smit, & Davies, 2009; Roca & Galdón, 2010; Sloboda, Kamler, Bulantová, Votýpka, & Modrý, 2007). Generally, they are transmitted by ticks of the genus *Amblyomma* (Baneth et al., 2003; Vilcins, Ujvari, Old, & Deane, 2009), which occur at high density in Wolf volcano (Schatz, 1991). However, *Hepatozoon* was also PCR‐detected in marine iguana blood extracted from the mosquito *Aedes taeniorhynchus* (Bataille et al., 2012), suggesting that transmission (the dynamics of which are still unclear) may occur via more than one vector. Since 2005 (when we started investigating *CM*), tick abundance at Wolf volcano has been steadily high. A comparably high density of ticks affecting iguanas is not found anywhere else in Galápagos and high tick density is associated with high prevalence of *Hepatozoon* at Wolf volcano (Fulvo, 2010), suggesting that, in Galápagos, ticks play a major role in the transmission of *Hepatozoon* to iguanas.

Despite the fact that very little is known about the prevalence and effects of hemogregarines on reptile hosts (Manwell, 1977; Schall, 1986; Sorci, 1995), pathology associated with *Hepatozoon* spp. infection is increasingly recognized (Wozniak, Kazacos, Telford, & McLaughlin, 1996). They seem capable to provoke significant inflammatory responses (Stacy, Alleman, & Sayler, 2011; Wozniak & Telford, 1991) and diseases such as hemolytic anemia and blood cell abnormalities, resulting in immunosuppression (Telford, 1984).

Previous studies of the relationship between ecto/hemoparasites and CORT in reptiles returned contradictory results because of the many causal relationships linking glucocorticoid levels to different independent factors. For this reason, experimental studies aimed at discriminating cause and effect have been increasingly performed. Given the conservation concern of the focal species, we were not allowed to use practices such as artificial infection or hormonal manipulations, considered invasive by the Galápagos National Park, the governmental authority that administrates biodiversity in Galápagos. Nevertheless, we used a comparative approach, although correlative, to study the role of parasites on endocrine activity, accounting for a factor related to CORT: reproductive state. In fact, according to the energy mobilization hypothesis that describes glucocorticoids increasing during period energetically demanding such as the gravidity (Romero, 2002), we hypothesized an increase in CORT levels in reproductive animals.

Thus, under the classical hypothesis that high parasite load is often associated with elevated glucocorticoid levels (Maier & Watkins, 1999), we tested whether CORT plasma levels increase with the intensity of infection by ectoparasites and/or *Hepatozoon* using two approaches. We first investigated CORT and parasite load in the two species at Wolf volcano (W), with females at different reproductive state. Although a replication of a site analogous to Wolf volcano would be recommendable, such a condition cannot be met in Galápagos. Thus, in a second approach, we attempted at a cross‐site comparison by comparing two populations of *CS* exhibiting different levels of parasite infection (highly parasitized versus non‐parasitized). To do so, we selected a non‐parasitized, and at a non‐reproductive‐state population of *CS* (Bahia Urbina, BU). Of course, by selecting a different site, we might introduce local confounding factors that may affect CORT levels. Aware of this possible bias, we considered BU the best reference as it is the *CS* population geographically closest to Wolf volcano, in Isabela Island. As additional peripheral symptoms that characterize infection and correlate with it, we also investigated the heterophils/lymphocytes (H/L) ratio, commonly used as hematologic marker of stress in several reptile species (Duggan, 1981; Moberg, 1985; Xuereb, Row, Brooks, MacKinnon, & Lougheed, 2012).

## 2 MATERIALS AND METHODS

### 2.1 Ethic statement

Animal manipulation and blood sampling were performed according to a protocol that minimized animal stress, in accordance with the European Community guidelines and with the approval of the Galápagos National Park. Samples were exported and imported under the CITES permits 101/BG and IT/IM/2015/MCE/01711, respectively.

### 2.2 Field sites and sampling

**Figure 2:**
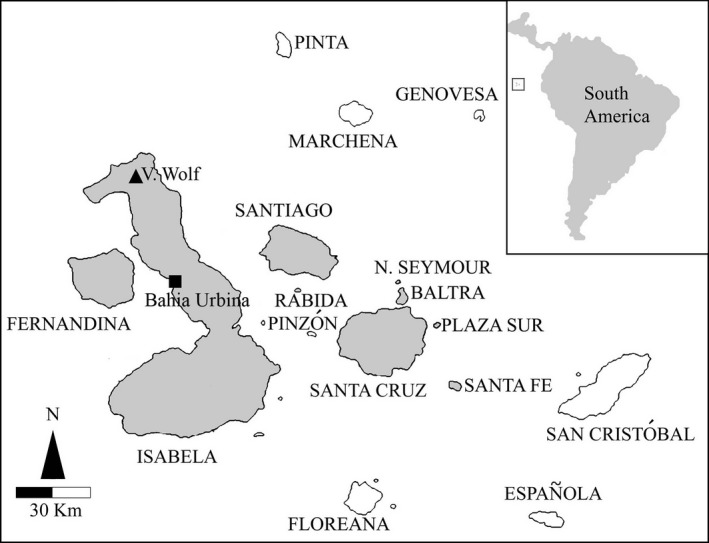
Galápagos Islands. The islands where *Conolophus* occurs or has occurred in historic times are in grey. The triangle indicates the volcano Wolf, the square indicates the coastal area Bahia Urbina, where samples were collected

The study was conducted in two different areas of Isabela Island: the Wolf volcano, the highest peak (1,707 m) in the Galápagos archipelago located on north side of the island, and Bahia Urbina, a coastal area situated on the west side (Figure 2).

Iguana's blood samples were collected in Wolf volcano in July 2010, June 2012, and 2014. In Bahia, Urbina samples were collected in June 2014. Tick load was recorded in 2012 and 2014, by counting ticks (all life‐cycle stages) in the axillar and gular areas, mostly impacted in iguanas.

During all field sessions, 2 ml of blood was collected from the caudal vein of each individual using a 5‐ml heparinized syringe. Blood samples were collected within 3–5 min from capture, under the assumption that this represent a sufficiently short time for CORT levels to represent baseline concentrations (Romero & Romero, 2002). Previous studies have shown that this time interval is sufficiently short to prevent that plasma levels of CORT be biased by capture stress (Cash, Holberton, & Knight, 1997; Romero, 2004; Romero & Wikelski, 2001; Sapolsky et al., 2000; Tyrrell & Cree, 1998; Wingfield et al., 1997).

We placed approximately 10 microliters of blood on the top of a slide and created a smear. Blood smears were air‐dried. Blood samples were placed on ice immediately after collection and later centrifuged for 2 min at 135 g to separate plasma. Plasma was kept at −10°C while in the field and then stored at −80°C until it was processed. Each iguana was weighed and snout‐vent length (SVL) was measured. The body condition index (BCI) was then estimated as the ratio of body mass/snout‐vent length (SVL)^3^ × 10^6^ (the ratio was multiplied by 10^6^ to reduce the number of decimals). This index has been already used for iguana species (Costantini et al., 2009; Laurie, 1989; Romero & Wikelski, 2001; Wikelski & Trillmich, 1997). For each female, we determined the number of eggs, egg size, and the stage of development of follicles using a Sonosite portable ultrasound machine (FUJIFILM SonoSite, Inc.) as in Gentile, Marquez, Tapia, and Izurieta (2016). We determined the reproductive status of each female, differentiating between reproductive (egg‐carrying) and non‐reproductive (without eggs) females. We distinguished different reproductive stages: stage “a,” females showing follicles with eggs of homogenous, spherical, and small dimensions; stage “b,” females with larger, yet not fully formed, unshelled eggs; stage “c,” females with large, fully formed, shelled eggs; stage “d,” females carrying no visible eggs inside follicles (Onorati et al., 2016).

### 2.3 Hematologic analysis

Blood smears were stained following the Romanowsky method, with modifications (Work, Raskin, Balazs, & Whittaker, 1998) to later count white blood cells (WBCs). We counted a total of 100 leukocytes, classified as granulocytic leukocytes as heterophils, eosinophils and basophils, and agranulocytes as monocytes and lymphocytes (Arikan & Çiçek, 2014), and calculated the heterophil to lymphocyte ratio (H/L).

We determined the parasitemia recording the number of erythrocytes infected by *Hepatozoon* (so far the only known hemoparasite infesting *Conolophus* spp., Fulvo, 2010) observed in 20 min, the time required to totally analyze approximately 10,000 erythrocytes and considered sufficient to obtain information about the intensity of infection (Valkiūnas, Iezhova, Križanauskienė, Palinauskas, & Bensch, 2008). Blood smears were scanned by the same investigator, at the same pace. If no hemoparasites were observed after this time, the individual was classified as uninfected. Trial sessions were blindly conducted prior to data collection to ensure consistency and repeatability.

### 2.4 Hormonal analysis

We determined plasma levels of CORT by competitive enzyme‐linked immunosorbent assays (ELISA). All ELISA immunoassays were performed at the Laboratory of Clinical Biochemistry (Tor Vergata University Hospital). On the whole, for analyzing CORT of all blood samples, we used kits ELISA (KA0468) pre‐coated with a polyclonal antibody. We used 10 μl of plasma diluted with 90 μl of assay buffer. The detection limit was established to be 0.28 ng/ml. The intra‐assay variation was 4.1% and the inter‐assay variation 10.1%.

All samples were assayed in duplicate and randomly distributed between plates. All assays were performed according to the instructions of the kit manufacturers.

### 2.5 Statistical analysis

We used STATISTICA 8 package for Windows, and Past version 3.07 for MAC.

Log‐transformed values of all hormonal and hematologic parameters were used to obtain normal distributions. We used one‐way ANOVA with Tukey's HSD (Honest Significant Difference) post hoc pairwise comparisons to analyze differences in parasitemia and CORT plasma levels among years.

We tested for statistical differences of parasitemia, body condition index and H/L ratio between infected and uninfected and among sexes with unpaired Student's *t* test. *F* tests were run to test for equal variances. Student's *t* test were performed accordingly, as implemented in Past ver 3.14 (Hammer, Harper, & Ryan, 2001).

Generalized linear models (GLZs) with an identity‐link function were performed to evaluate which factors better explained the variation of CORT plasma levels. Females and males were analyzed separately in GLZ models, as in vertebrates sex differences in adrenocortical activity have been described (Kirschbaum, Wüst, & Hellhammer, 1992; Kudielka & Kirschbaum, 2005). For CORT, to ensure proper evaluation of interactive effects, for both sexes we considered two different models. Model 1 included only *CM* and *CS* from Wolf volcano, whereas Model 2 did not include *CM* and included both populations of *CS* (W+BU).

For males and females, all models included species (or site for the model including only *CS* populations) as categorical factor and body condition index, parasitemia, ticks number, and H/L ratio as covariates. For females, reproductive state was also included as categorical factor (yes or no). We tested also for the interaction between species and reproductive state.

## 3 RESULTS

### 3.1 Corticosterone and parasitemia

Overall, for *CM*, we analyzed 82 individuals whereof 66 were infected (80%); for *CS* we analyzed 61 individuals whereof 45 were infected (74%).

Parasitemia of both species is shown in Table 1. Overall, parasitemia was higher in *CS* than in *CM* (*t *=* *4.3; *p = *.0001).

**Table 1:** Parasitemia of *C. subcristatus* (*CS*) and *C. marthae* (*CM*) from Wolf volcano (W) and *C. subcristatus* from Bahia Urbina (BU)

Sex	Sp./Year	N	Infectedby *Hep*.	Infected by ticks	Parasitemia			Ticks		
					Mean	SE	Median	Mean	SE	Median
Females	*CM* (W)									
	2010	9	8	N/a	30	10.8	15	‐	‐	‐
	2012	11	9	11	44.4	24.5	14	35.7	6.5	30.5
	2014	18	13	18	27.7	13.3	9	47	9.4	39
	*CS* (W)									
	2010	4	3	N/a	98.2	43.6	101.5	‐	‐	‐
	2012	11	8	11	59.4	34.5	11	45	6.8	38
	2014	15	12	14	46.8	20.8	17	53.3	4.7	54
Males	*CM* (W)									
	2010	8	7	N/a	22.1	8.1	14	‐	‐	‐
	2012	11	8	11	17.1	6.6	11	38.4	5	37
	2014	23	19	23	8.5	3.1	3	61	6.2	52
	*CS* (W)									
	2010	4	3	N/a	48	65.8	19.6	‐	‐	‐
	2012	11	7	11	28	33.5	12	52.2	6.1	57
	2014	12	10	12	41	25	3.5	49.7	5.2	43
Females	*CS* (BU)									
	2014	15	1	1	1.6	1.6	0	0.06	0.06	0
Males	*CS* (BU)									
	2014	12	1	3	0.08	0.08	0	0.33	0.2	0

For both species, considering males and female separately, no difference in parasitemia among years was found (for all *p *>* *.05). No difference in parasitemia emerged between sexes (*CM*:* t *=* *1.7, *p *=* *.08; *CS*:* t *=* *0.9, *p *=* *.3). In both species, we did not observe a significant difference between CORT plasma levels of infected and uninfected individuals, after pooling sexes and years (*CM: t *=* *0.7, *p *=* *.5; *CS*:* t *=* *1.2, *p *=* *.8). Parasitemia did not explain the variance of CORT plasma levels in either males (Wald = 0.005, *df* = 1, *p *=* *.94) or females (Wald = 0.74, *df* = 1, *p *=* *.39) on Wolf volcano (Model 1) nor considering only populations of *CS* (W+BU, Model 2; females: Wald = 0.02, *df* = 1, *p *=* *.88; males: Wald = 2.74, *df* = 1, *p *=* *.09). Only in *CS* from Wolf volcano, a positive correlation between H/L ratio and parasitemia emerged (*r *=* *0.27; *p *=* *.04). In both species, BCI did not differ between infected and uninfected individuals (*CM*:* t *= −0.7, *p *=* *.5; *CS*:* t *=* *0.8, *p *=* *.4) (Table 2).

**Table 2:** Body condition index (BCI) of individuals infected and uninfected by *Hepatozoon* (mean, standard error, and median are reported)

Sex	Sp./Year	Infected			Uninfected		
		Mean	SE	Median	Mean	SE	Median
Females	*CM* (W)						
	2010	51.2	2.5	52.8	45.2	0	45.2
	2012	53.7	3.6	49.8	47.2	0.7	47.2
	2014	54.8	2.2	52.2	59	4.2	53.5
	*CS* (W)						
	2010	45.6	5	41.1	40.4	0	40.4
	2012	50.2	3.1	46	49.6	4.8	48.8
	2014	49.8	1.8	50.5	56.5	4.7	55.4
Males	*CM* (W)						
	2010	51.5	3.8	51	56.5	0	56.5
	2012	49.6	2	49.1	56	5.1	59.6
	2014	55.7	2.1	53.1	55.9	2.5	56.7
	*CS* (W)						
	2010	50.2	3.8	46.7	48.4	0	48.4
	2012	49.9	2.9	47.1	59.5	3.5	58.4
	2014	59.4	3.3	59.5	55	1.1	55.3
Females	*CS* (BU)						
	2014	69.4	0	69.4	69.1	3.9	67.9
Males	*CS* (BU)						
	2014	76.5	0	76.5	65.6	2.6	64.6

### 3.2 Corticosterone plasma levels in females

The reproductive status of females is reported in Table 3.

**Table 3:** Reproductive states of females living on Wolf volcano a) females showing follicles with eggs of homogenous, spherical, and small dimensions; b) females with larger, yet not fully formed, unshelled eggs; c) females with large, fully formed, shelled eggs; d) females carrying no visible eggs inside follicles

Species	Year	Reproductive state				Tot
		a	b	c	d	
*CM*	2010	0	0	3	6	9
	2012	0	1	0	10	11
	2014	3	0	1	14	18
*CS*	2010	0	0	0	4	4
	2012	0	0	10	1	11
	2014	1	1	8	5	15

In females of *CM*, CORT plasma levels ranged from 0.16 to 74.11 ng/ml, whereas in *CS* it ranged from 0.22 to 158 ng/ml (mean and medians are reported in Table 4).

**Table 4:** Corticosterone plasma levels in *C*. *marthae* and *C. subcristatus*

Sex	Species	N	Corticosterone ng/ml		
	Year		Mean	SE	Median
Females	*CM* (W)				
	2010	9	3.8	2.8	0.9
	2012	11	13.4	6.7	4
	2014	18	8.9	1.4	0.6
	*CS* (W)				
	2010	4	2.2	0.8	1.9
	2012	11	75.1	19.5	105
	2014	15	16	7.7	5.1
Males	*CM* (W)				
	2010	7	31.1	27.9	2.7
	2012	11	1.1	0.4	0.5
	2014	23	1.9	0.7	0.7
	*CS* (W)				
	2010	4	5.4	3.9	2.1
	2012	11	20.5	9.7	10.6
	2014	16	13.7	9.4	0.8
Females	CS (BU)				
	2014	15	16.4	7.3	6.8
Males	CS (BU)				
	2014	12	64.9	18.6	38.0

**Figure 3:**
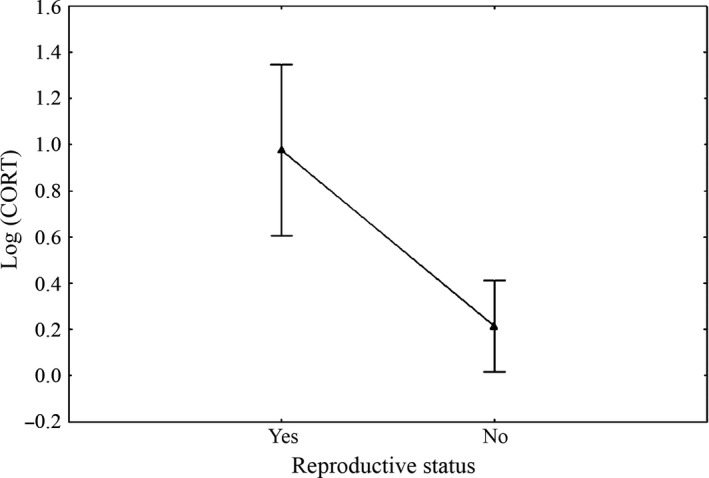
Corticosterone and reproductive status. Vertical bars denote 95% confidence intervals

**Figure 4:**
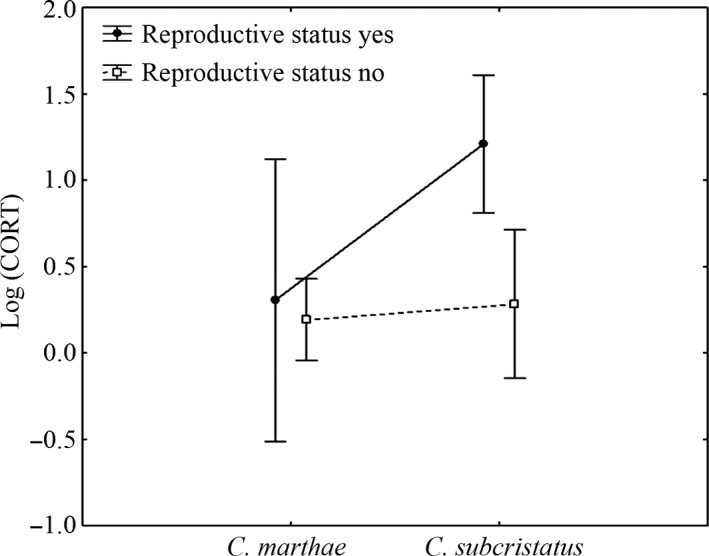
Corticosterone variation in relation to the interactive effect between species and reproductive status. Vertical bars denote 95% confidence intervals

For both species, we observed statistically significant differences among years (*CM*:* F *=* *6.3; *p *=* *.004; *CS*:* F *=* *5.9 *p *=* *.007). The CORT levels showed a maximum in 2012 for *CM* (Tukey's HSD test; *p*_*2012–2010*_ **= .03, *p*_*2012–2014*_ *= *.007), whereas for *CS* we recorded a higher concentration in 2012 than in 2010 (*p*_*2012–2010*_ =.009). On the volcano, the variance of CORT levels was explained only by reproductive state (Wald = 7.89, *df* = 1, *p *=* *.005, Figure 3) and by its interactive effect with species (Wald = 4.55, *df* = 1, *p *=* *.03, Figure 4). CORT variance was not statistically explained by species (Wald = 1.534, *df* = 1, *p *=* *.215), BCI (Wald = 1.668, *df* = 1, *p *=* *.196), tick number (Wald = 2.92, *df* = 1, *p *=* *.087), and H/L although in this case a positive CORT‐H/L relationship was marginally non‐significant (Wald = 3.54, *df* = 1, *p *=* *.059).

No variable explained the variance of CORT levels in females of *CS* (W+BU) (all *p *≥* *.09).

### 3.3 Corticosterone plasma levels in males

**Figure 5:**
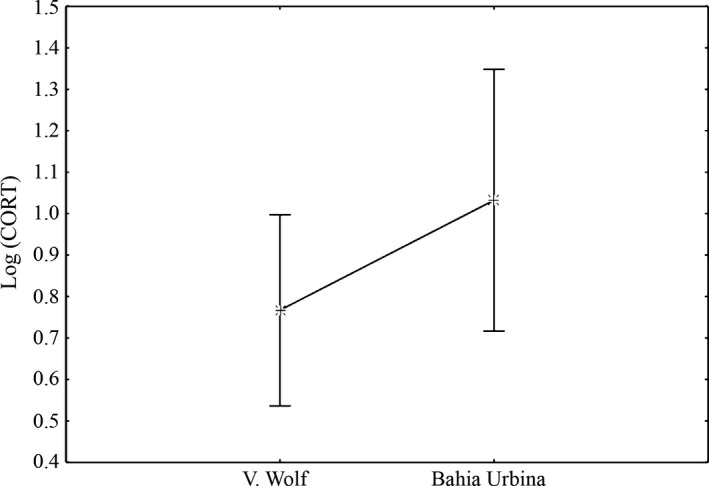
Corticosterone variation in *Conolophus subcristatus* in relation to site. Vertical bars denote 95% confidence intervals

In males *CM*, CORT plasma levels ranged from 0.21 to 13.9 ng/ml, whereas in *CS* it ranged from 0.19 to 153 ng/ml (mean and medians are reported in Table 4). In both species, no differences among years were detected (*CM*:* F *=* *1.5, *p = *.2; *CS*:* F *=* *2.3, *p *=* *.1). On Wolf volcano, only H/L ratio showed a clear effect on CORT with a positive relationship (Wald = 7.35, *df* = 1, *p *=* *.007). Site was the only explanatory variable of CORT variance for *CS* populations (Wald = 10.35, *df* = 1, *p *=* *.001), with males from Bahia Urbina showing higher CORT plasma levels (Figure 5). CORT variance in *CS* males was not statistically explained by tick number (Wald = 0.11, *df* = 1, *p *=* *.741) or H/L although a positive CORT‐H/L relationship was marginally non‐significant (Wald = 3.64, *df* = 1, *p *=* *.056).

## 4 DISCUSSION

In this study, we used CORT plasma levels to investigate possible impacts of ticks and *Hepatozoon* on the stress physiology of Galápagos land iguanas. Despite the fact that an inverse relationship between the level of hemoparasite/ectoparasite infection and glucocorticoid plasma concentration was previously found in iguanas (Hanley & Stamps, 2002), we did not find evidence in support of a such a relationship in the populations of *C. marthae* and *C. subcristatus* from Wolf volcano. In fact, in both species from the site, we did not observe significant differences in baseline CORT levels or body condition index between hemo‐parasitized and non‐hemo‐parasitized individuals. Similarly, we did not observe a significant correlation between CORT levels (or body condition index) and the number of ticks or parasitemia. We cannot rule out the hypothesis that the level of hemo‐parasitism at Wolf volcano might not meet the required threshold to activate an endocrine response. This would be consistent with the view that hemogragarines do not necessarily cause disease. In fact, clinical signs including lethargy, open‐mouth breathing, weight loss, and dehydration may be observed in immune‐compromised individuals (Nardini, Leopardi, & Bielli, 2013). However, it is also possible that the hemo‐ and non‐hemo‐parasitized individuals show similar low levels of CORT because they are both highly impacted by the chronically high tick density that affects both hemo‐ and non‐hemo‐parasitized individuals. Wolf volcano is the site where ticks occur at the highest density in Galápagos islands, with the minimum tick load per individual being in the order of tens. This load could be high enough to mask a correlation between CORT levels and number of ticks. Possibly, these results can be considered in the light of the immune redistribution hypothesis (Braude, Tang‐Martinez, & Taylor, 1999; Maier & Watkins, 1999), which would allow to explain low levels of CORT in highly tick‐infected iguanas (Wolf volcano) as a way for the immune system to focus on internal parasites such as hemogregarines (Hanley & Stamps, 2002). In fact, highly hemo‐parasitized *CS* from Wolf volcano did show significant alteration in some measures of immune function, with an increase in H/L ratio being found. Although CORT plasma concentration was not associated with parasitemia or tick load at Wolf volcano, some evidence of a positive relationship between CORT and H/L emerged from our data although not fully statistically supported. A review of the positive association between CORT and H/L may be found in Davis, Maney, and Maerz (2008).

The two most abundant white blood cells (H, L) are expected to inversely respond to *Hepatozoon* infection when immune system is activated against hemogregarines (Xuereb et al., 2012). Thus, in *CS*, an activation of immune system especially in phagocytic cells emerged, as reported for many parasitized vertebrates (Davis, Cook, & Altizer, 2004; Lobato, Moreno, Merino, Sanz, & Arriero, 2005). The observed difference in CORT levels between Wolf volcano and Bahia Urbina, which was not parasitized and showed CORT levels higher than Wolf populations, is consistent with this view and with Hanley and Stamps (2002) who found association between low levels of CORT and high infection in free‐living *Ctenosaura similis*. However, we admit that other factors (reproductive state, altitude, tourism, etc.) may potentially be associated with CORT levels at Bahia Urbina.

Although CORT did not correlate with parasites or body condition index, it positively correlated with reproductive condition in females. CORT levels appeared elevated in females carrying eggs (small, not fully formed or fully formed) in both land iguana species. Also in marine iguanas, CORT was mostly elevated during the gestation and nesting period before eggs were laid and it declined significantly immediately after egg‐laying (Rubenstein & Wikelski, 2005). In our study, the observed increase in CORT in reproductive females could reflect the energetic demands of reproduction (Wingfield, 1988). This would be consistent with the energy mobilization hypothesis by Wingfield and Ramenofsky (1999), according to which CORT concentrations are highest during periods that require energy supply. This evidence is important, as it would indicate that populations at Wolf volcano are capable to mount an adequate endocrinologic response to mobilize the energy required for reproduction.

Although all non‐reproductive females showed similar CORT baseline levels without a difference between species, in reproductive females we observed a difference in favor of *CS*, in which most individuals (90%) showed mature eggs. Thus, in case no biologic difference in the physiological process of CORT production between the two congeneric species exists, the CORT increase in reproductive *CS* females with fully formed eggs could also be associated with the metabolic change specifically required for egg development, as already described in other reptiles (Wilson & Wingfield, 1992). Indeed, generally a positive association between reproductive state and glucocorticoids level has been observed for many egg‐laying vertebrates (Silverin & Wingfield, 1982; Wack, Fox, Hellgren, & Lovern, 2008; Wilson & Wingfield, 1992). Many studies (Grassman & Crews, 1989; Moore & Jessop, 2003; Taylor, DeNardo, & Jennings, 2004) demonstrated that elevated CORT plasma levels facilitate reproduction by mobilizing energy stores for egg production processes such as vitellogenesis, oocyte maturation, and ovulation. This hypothesis awaits further investigation.

## CONFLICT Of INTEREST

None declared.
